# Supplementary material for: Single-Cell RNA Sequencing of Baseline Immune Profiles After Third Vaccination Associated with Subsequent SARS-CoV-2 Infection in Naïve Individuals
Source: Int J Mol Sci. 2025 Apr 8;26(8):3494. doi: 10.3390/ijms26083494 (PMC12027237; doi:10.3390/ijms26083494)
Supplement: Supplementary file 1 [file ijms-26-03494-s001.zip › ijms-3548205-Supplementary Information.pdf]

Supplementary information

Supplementary Table S1. The statistics of scRNA-seq raw data.

Sample	Number of cells	Median transcripts per cell	Median genes per cell	Reads mapped to genome
**Protected_1**	6,167	1,342	415	36,885,617
**Protected _2**	4,351	1,230	373	25,343,970
**Protected _3**	7,820	5,190	1,910	109,989,376
**Infected_1**	7,214	2,589	1,049	58,487,136
**Infected_2**	5,258	4,404	1,650	61,976,207
**Infected_3**	6,314	1,178	346	32,879,812
**Infected_4**	9,694	5,229	1,916	138,732,245

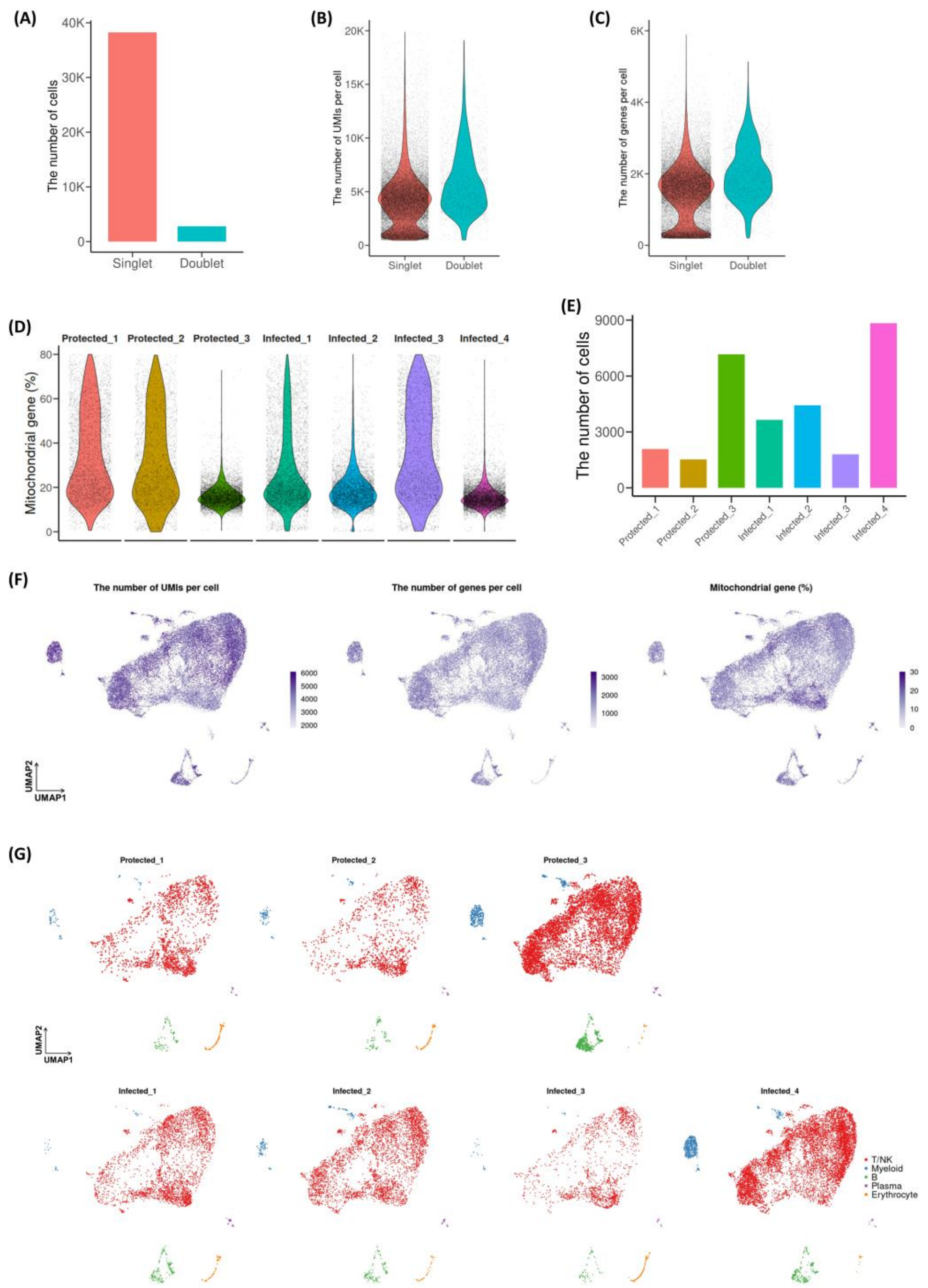

Supplementary Figure S1. Quality control of scRNA-seq data. (A) The number of singlet and doublet cells inferred by DoubletFinder. (B, C) Violin plots of the number of UMIs (B) and the number of expressed genes (C) in the singlet and doublet cells. (D) Violin plots of the percentage of expressed mitochondrial genes in each sample. (E) The number of cells in each sample after the filtering for quality control (only singlet cells with the percentage of mitochondrial genes < 30%) (F) UMAP plots colored by the number of UMIs (left), the number of expressed genes (center), and the percentage of mitochondrial genes (right) (G) UMAP plots of each sample.
